# A Facile Solution Engineering of PEDOT:PSS-Coated Conductive Textiles for Wearable Heater Applications

**DOI:** 10.3390/polym13060945

**Published:** 2021-03-19

**Authors:** In Su Jin, Jea Uk Lee, Jae Woong Jung

**Affiliations:** Integrated Education Institute for Frontier Science & Technology (BK21 Four), Department of Advanced Materials Engineering for Information and Electronics, Kyung Hee University, Yongin-si 446-701, Gyeonggi-do, Korea; jininsu1022@gmail.com

**Keywords:** electronic textiles, PEDOT:PSS, Joule heaters

## Abstract

To enable highly conductive electronic textiles (E-textiles), we herein demonstrate a simple solution treatment of poly (3,4-ethylenedioxythiophene): poly (styrene sulfonate) (PEDOT:PSS)-coated textiles by dimethyl sulfoxide (DMSO) and methanol. The subsequent solution engineering of DMSO and methanol not only enhances crystallization of PEDOT chains but also the contact for PEDOT:PSS to the fibers. Additionally, the methanol dipping effectively removes the insulating PSS part from the conductive PEDOT chains, which contributes to subsequently reduced sheet resistance of less than 3 Ω/sq of the conductive textiles. Joule heating property of the highly conductive textiles achieves the maximum temperature with the temperature reaching 133 °C at a low applied voltage of 3 V within 20 s, which promises highly conductive E-textiles as multi-functional wearable heater applications.

## 1. Introduction

There has been growing demand for electronic textiles (E-textiles) that combine clothes and multi-functional electronics [1,2,3]. E-textiles are intrinsically designed to be equipped with wearable and portable electronic technologies, and their comfortable and light-weight characteristics afford unsurpassed electronic applications in the field of healthcare, energy, bioelectronics, and smart clothing [4,5,6,7,8,9,10,11,12,13,14]. Recent advances in E-textiles have developed numerous materials/engineering technologies for flexible conductors, most of which focus on incorporating conductive materials such as metallic nanomaterials, carbon materials, or thin metal layers in elastomers [15,16,17]. Despite their excellent electrical properties, the metallic nanomaterials have limited degrees of stretchability and flexibility, which hamper practical applications in wearable electronics. Additionally, the metal/carbon composite materials are not only too rigid or fragile to withstand large deformation but are also as thick as >1 μm to reach enough electrical conductivity. As an alternative, conducting polymers have emerged as promising conductive materials for the development of E-textiles because their intrinsic flexibility, light weight, low cost, conformability with the human body, and potential of solution processing. Poly (3,4-ethylenedioxythiophene): poly (styrene sulfonate) (PEDOT:PSS) is one of the most well-established conducting polymers [18,19,20]. PEDOT:PSS material has the advantages of high mechanical flexibility, biocompatibility, solution processability, thermal stability, and long-term stability when compared to other conductive polymers. In pursuit of the enhanced electrical conductivity for PEDOT:PSS, a variety of additives or treatments have been reported, and the conductivity can reach to metallic level (up to >1000 S cm^−1^) [21,22,23,24,25]. Moreover, PEDOT:PSS is commercially available in aqueous solution and readily processable on textile-based substrates with low-cost methods in large areas, which offers great potential to printable electronics in large areas via facile printing technologies [26].

The PEDOT:PSS is a simple mixture of PEDOT and PSS in which PEDOT chains are coupled with the ionic PSS chains, resulting in the doped polymer dispersion. Thus, PEDOT:PSS can not only be well dispersed in water, but also enhance the electrical conductivity. In the commercially provided PEDOT:PSS, PEDOT and PSS are mixed in the weight ratio of ~1:2.5 to balance the charge with counter ions each other, so it exhibits relatively low conductivity (typically less than 1 S cm^−1^) due to excess PSS present at the film surface. Much effort has been made to improve the conductivity of PEDOT:PSS, including additive engineering or the post treatments. As an additive, the organic compounds such as polyols, surfactants, polar solvents, and zonyl have been added in the PEDOT:PSS solution [27]. Among them, the polar solvents such as dimethylsulfoxide (DMSO), dimethylformamide, and *n*-methyl-2-pyrrolidone DMSO have been exceptionally effective as a secondary dopant to enhance conductivity in thin film state, because these additives rearrange the polymer chains and surface structure of PEDOT:PSS. However, the additive engineering of PEDOT:PSS in textile substrates is confronted with uniformity and stability problems because the aqueous solution easily penetrates in the woven structure of fabric, and thus appropriate contact of PEDOT:PSS to the fibers could be deteriorated, which cannot generate ideal electrical pathway for electrons. In this regard, it is imperative to find more facile but effective approaches for preparing the large-area E-textile with high uniformity and excellent electronic properties.

Here, we present a facile strategy for fully printable multi-functional E-textiles based on PEDOT:PSS. We suggest a simple solvent treatment to enhance the electrical conductivity of PEDOT:PSS/DMSO-coated E-textile by dipping it in methanol/DMSO solution. The corresponding PEDOT:PSS film was highly conductive with substantially decreased sheet resistance from 66.89 to 2.65 Ω/sq. The Joule heating properties of the highly conductive E-textiles were discussed to clearly demonstrate the potential application of the E-textiles in textile heaters. The solvent-treated E-textiles exhibited better Joule heating properties achieving high temperature at low operation power in quick response time. The E-textiles also exhibited good susceptibility on the cyclic elongations, wash tests, and long-term operation, which revealed great potential of the PEDOT:PSS-based E-textiles for practical applications in wearable electronics.

## 2. Materials and Methods

### 2.1. Preparation of PEDOT:PSS Composite Solutions

The commercially available microfiber fabric (Welcron, nylon/polyester splitting yarn) was used as a substrate for the fabrication of the conductive textiles. The surface of fabric substrate was cleaned with detergent and then dried under dry air. The PEDOT:PSS (PH1000, Heraeus Clevios, Hanau, Germany) was diluted with deionized water (1:1 in volume), and then 4.5 wt.% of DMSO was added to the PEDOT:PSS dispersion. The solution was then vortexed for 15 min and then placed in room temperature for 15 min before coating.

### 2.2. Preparation of E-Textiles

The PEDOT:PSS solution filtered with a nylon syringe filter (pore size = 0.2 μm) was dripped onto the fabric substrate (5.0 × 5.0 cm^2^). The solution was instantaneously spread out over the textile by penetrating into the fabric pores, so the first sample (sample 1) of the E-textiles was completed after drying by heat gun. The second sample (sample 2) was prepared by immersing sample 1 into the methanol/DMSO solution (4.5 wt.% of DMSO in methanol) at room temperature for 7 min, followed by heat-gun drying. Two side edges of the sample were coated with silver paste (Sigma-Aldrich, St. Louis, MO, USA) and then placed at room temperature for drying.

### 2.3. Characterizations

The surfaces of the E-textiles were observed by the optical microscope (BX53M, Olympus, Tokyo, Japan). The nanostructure of surfaces and cross-sectional morphologies were further observed by field-emission scanning electron microscopy (FE-SEM, Hitachi, Tokyo, Japan) equipped with energy-dispersive spectroscopy (EDS). The crystalline properties of the samples were measured by an X-ray diffraction (XRD) instrument (Miniflex 300, Rigaku, Tokyo, Japan). The elemental structure of the samples was investigated by X-ray photoelectron spectroscopy (XPS) (K Alpha, Thermo Science, MA, USA) using a MgKα/AlKα dual anode X-ray source. The sheet resistance of the samples were measured by four-point probe station (Ossila, Sheffield, England) equipped by a four probes spacing of 1.27 mm between the probes (0.48 mm probe diameter). A bias of ~20 mV was applied through the probes and the current level of 10–15 mA was recorded during the measurement. The Joule heating characteristics of the E-textiles were measured by applying DC voltage to the silver contact electrode by a power supply (Keithley 2400, Tektronix, OR, USA). The temperature images and temperature distribution were measured using the thermal camera (ETS320, FLIR, OR, USA). The bending test of the samples was performed by the home-made linear motor system. The samples were kept in the ambient atmosphere (T~20 °C, RH~45%) in darkness and the sheet resistance of the samples was measured by four-point probe station.

## 3. Results

### 3.1. Preparation of PEDOT:PSS-Coated E-Textiles

**Figure 1:**
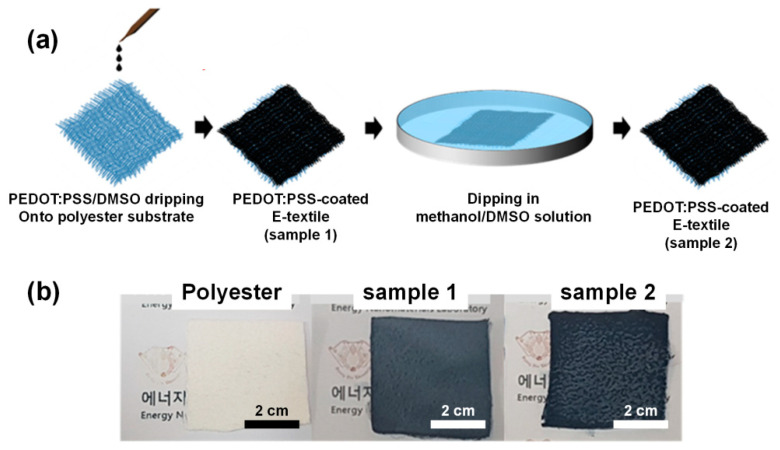
(**a**) Schematic illustration of the preparation of electronic textiles (E-textiles) and (**b**) the photographs of the E-textiles with a polyester textile substrate.

**Figure 2:**
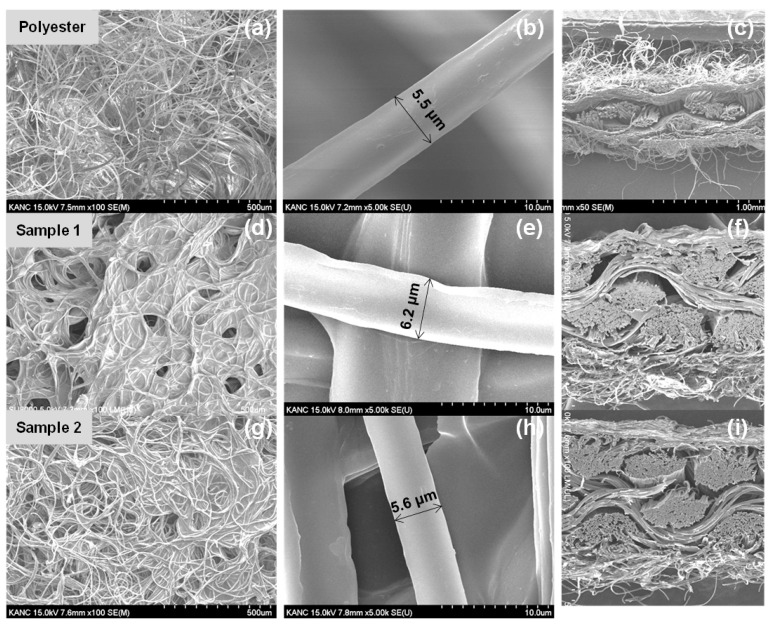
Scanning electron microscopy (SEM) images showing morphology of the pristine textile (**a**–**c**), sample 1 (**d**–**f**), and sample 2 (**g**–**i**) at low magnification (**a**), medium magnification (**b**,**c**) cross-sectional SEM images.

The fabrication procedure of PEDOT:PSS-coated E-textiles is described in Figure 1a. Two samples of E-textiles were prepared to compare the effect of solvent treatment on the electrical properties of the E-textiles. As shown in Figure 1b, the color of the sample became a little darker after dipping in the methanol/DMSO solution. In addition, sample 1 was smooth but rather hardened after drying, while sample 2 was softer than sample 1. The optical microscopies of the samples showed that the solution permeated into the textile and each fibrils were coated by PEDOT:PSS (Figure S1 in supplementary material). Figure 2 shows the nanostructure of the E-textiles analyzed by scanning electron microscopy (SEM). Sample 1 shows that the fibril surfaces of the E-textile were coated with PEDOT:PSS, but the excess solution was immobilized inside of the textile as the solution dried, and thus the solution filled the pores between the fibrils. However, the excess PEDOT:PSS was effectively removed by methanol/DMSO dipping, as confirmed by reduced fibril size of sample 2 rather than sample 1. (Figure 2b,e,h) The EDS was employed to further confirm the degree of PEDOT:PSS coating of the fabric substrate. The corresponding EDS images of PEDOT:PSS-coated E-textiles are displayed in Figure S2 [28]. The quantitative data of the EDS analysis for PEDOT:PSS/DMSO-coated textiles and PEDOT:PSS-coated textiles treated with methanol are shown in (Table S1 in supplementary material). It can be seen that PEDOT:PSS was well-coated on the individual fibril bundles over entire textile substrate, which was proved by well-distributed carbon, oxygen, and sulfur elements in the E-textiles. It is noted that the elemental mapping at the pores between fibril bundles of sample 2 were rather lower than sample 1, which also confirmed that the dipping effectively removed excess PEDOT:PSS in the E-textiles [29,30].

### 3.2. Electrical Properties of E-Textiles

**Figure 3:**
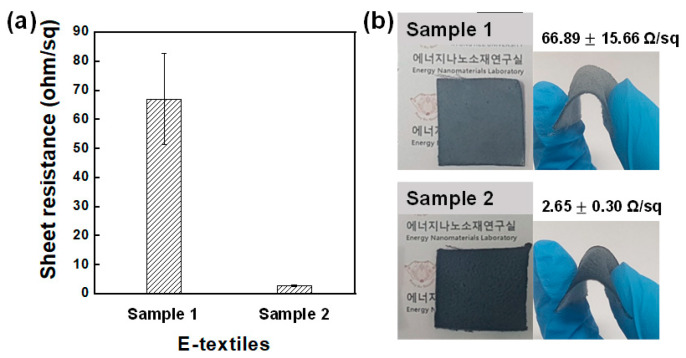
(**a**) Sheet resistance of E-textiles and (**b**) their photographic images.

Figure 3 compares the sheet resistance of E-textiles. The average sheet resistance of E-textiles prepared PEDOT:PSS/DMSO coating (sample 1) was 66.89 Ω/sq, and it was substantially decreased to 2.65 Ω/sq after dipping in methanol/DMSO. Since the excess PEDOT:PSS in the pores of the fabric substrate was removed, the substantially decreased resistance of the E-textiles (sample 2) was only explainable by new hypothesis. To investigate the reason for the increased conductivity, we compared the crystallinity of PEDOT:PSS films coated on the fabric substrates.

**Figure 4:**
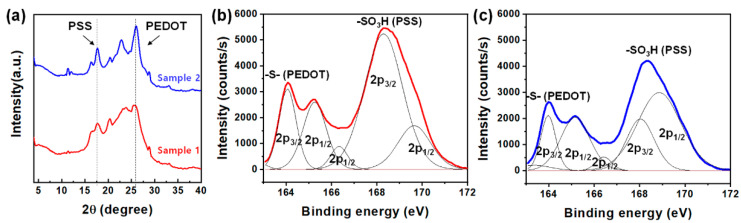
(**a**) X-ray diffraction (XRD) scans of E-textiles, (**b**) X-ray photoelectron spectroscopy (XPS) spectra of sample 1, and (**c**) XPS spectra of sample 2.

Figure 4a displays the diffractograms of PEDOT:PSS-coated E-textiles. For the PEDOT:PSS thin film, the low angle reflections at 2θ = 3.8° and 6.6° corresponded to the lamella stacking distance *d*(100) of two distinct alternate orderings of PEDOT and PSS, whereas the two high angle reflections at 2θ = 17.7° and 25.6° are indexed to the amorphous halo of PSS and the inter-chain planar ring-stacking distance of *d*(010) of PEDOT, respectively [31]. It is interesting that sample 2 exhibited more intense peak at 2θ = 25.6° as compared to sample 1, rather than 2θ = 17.7°. The XPS of the PEDOT:PSS films on the textile substrate was investigated to study the oxidation level of PEDOT films. As shown in Figure 4b,c, the S2p peaks of PEDOT:PSS were specified by two distinct types of sulfur atoms in the thiophene unit in PEDOT and the sulfonate group in PSS, respectively. The broad peak at higher binding energy was attributed to the S2p_3/2_ and S2p_1/2_ peaks of the sulfonate groups in PSS while the S2p_3/2_ and S2p_1/2_ doublet peaks were attributed to the sulfur atoms in PEDOT due to the oxidation of thiophene unit in the PEDOT chains [32]. The increased ratio of S2p in PEDOT to that of PSS in sample 2 indicated that the PSS chains were effectively removed by the solvent treatment (dipping in methanol/DMSO) [33]. It is suspected that the effective removal of PSS by solvent treatment also affected the self-assembly of PEDOT chains, as confirmed by XRD analysis (Figure 4a). Thus, the removal of insulating PSS would contribute to enhanced electrical conductivity of the PEDOT:PSS-coated E-textiles. In addition, the increased crystallinity of PEDOT chains also facilitated electron transport along the PEDOT:PSS films of the E-textiles [23,34].

**Figure 5:**
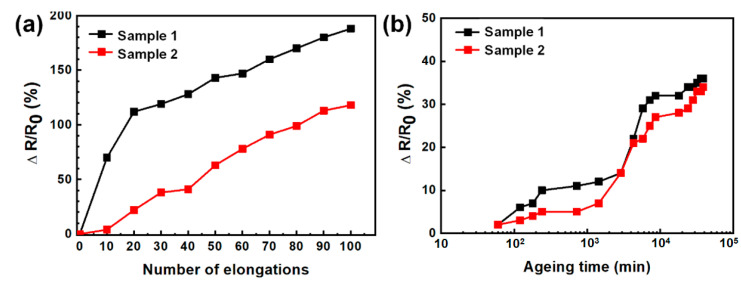
Sheet resistance variation of E-textiles under stretching (**a**) and ageing test (**b**).

The stretchability is a unique characteristic of E-textiles for application in wearable electronics. Figure 5a shows the variation of sheet resistance of E-textiles upon stretching cycles (10% strain). Sample 2 exhibited ~110 % increase in the sheet resistance, while sample 1 showed ~180% increase in the resistance after 100 cycles of stretching, so it was obvious that sample 2 was more susceptible to tensile stress than sample 1. The sheet resistance variation of E-textiles would be mainly affected by geometric changes in the deformed E-textiles upon tensile stress. In order to confirm the different behavior of two E-textiles under tensile stress, the geometric change of the PEDOT:PSS films were investigated by SEM. (Figures S3 and S4 in supplementary material). In the case of sample 1, the bundles of fibrils were divided into several parts and severe cracks of PEDOT:PSS film were formed after stretching. The disconnection between the PEDOT network and the broken fiber would affect sudden increase of sheet resistance. Therefore, it is suspected that the physical disconnection of PEDOT:PSS film is the main reason for the substantial increase in the sheet resistance of E-textiles during the stretching cycles. Unlike sample 1, most PEDOT:PSS was uniformly coated on each fibril in sample 2, so no obvious mechanical breakage or crack was observed after elongation. Thus, the solvent-treated PEDOT:PSS could suppress substantial loss in electrical conductivity under continuous mechanical stretching, which would be favorable feature for application in wearable electronics [35]. We then compared the evolution of the electrical resistance of two E-textiles in ambient atmosphere over the course of 40,000 min to assess the long-term stability. As shown in Figure 5b, the sheet resistance changed by not more than 10% for 2000 min, but it gradually increased by 36% and 34% for sample 1 and sample 2, respectively, after 4000 min in ambient storage. This result revealed an acceptable level of ambient stability of the PEDOT:PSS-coated E-textiles for practical applications.

**Figure 6:**
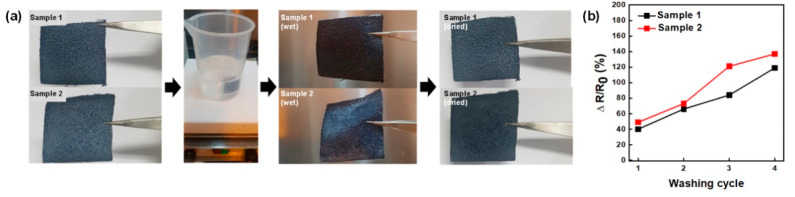
(**a**) Washing test and (**b**) sheet resistance ageing test of E-textiles.

To enable the practical use of the E-textiles, it should be able to endure the washing process. As shown in Figure 6a, we simply assessed the washing reliability of the E-textiles by immersing the samples in deionized water with a stirring at 1500 rpm for 30 min. The textiles were then dried by heat gun for 3 min between each washing cycle. The sheet resistance increased to 120% and 135 % for sample 1 and sample 2, respectively, after four washing cycles. (Figure 6b) The increase in the sheet resistance may have been due to oxidation of the PEDOT chain as the samples were contacted with water molecules. The sheet resistance increase of sample 2 was higher than sample 1, which would be due to the larger surface area of the PEDOT:PSS film in the E-textiles. As such, it is suspected that the PEDOT:PSS of sample 2 would dissolve or collapse upon contact with water, as the electrical conductivity is greatly reduced as compared to sample 1, which has an adverse effect on the practical use of the E-textile [36].

### 3.3. Joule Heating Properties of E-Textiles

We fabricated the textile heater using the Joule heating of the E-textiles. The Joule’s law described the correlation between resistance, applied voltage in a circuit, and the heat energy generated from the electric energy.

*P* = *V*^2^/*R* (1)

**Figure 7:**
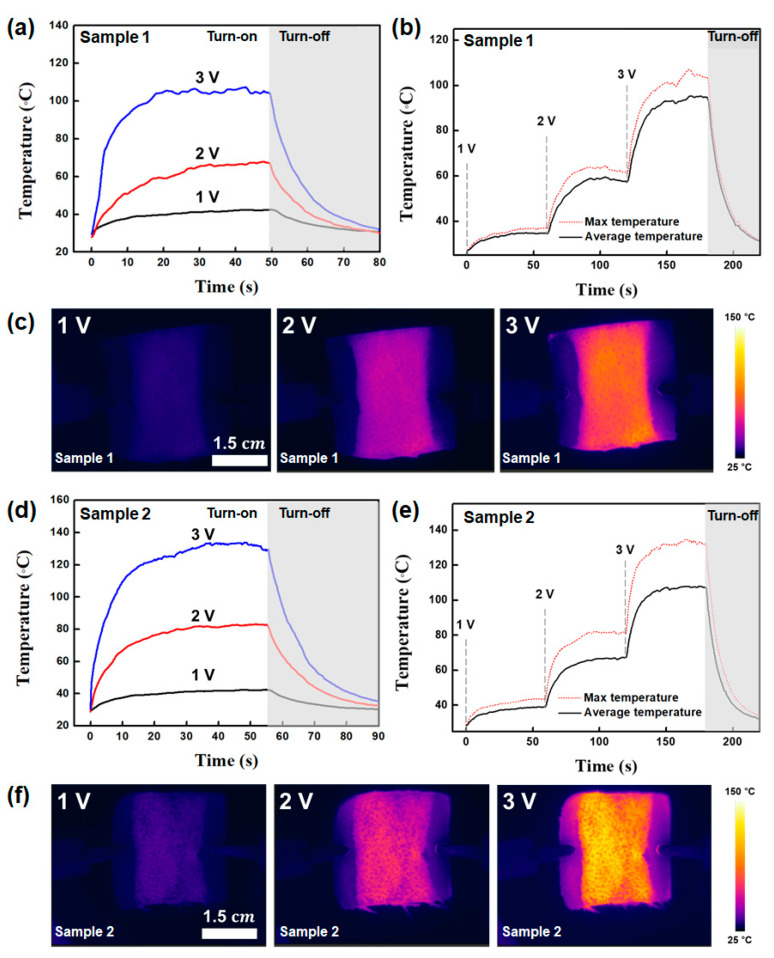
(**a**) Joule heating characterization, (**b**) maximum and average temperature, and (**c**) corresponding infrared images of sample 1 with an applied bias from 1 to 3 V. (**d**) Joule heating characterization, (**e**) maximum and average temperature, and (**f**) infrared images of sample 2 with a bias of 1 to 3 V.

The equation of Joule’s law constructs the power (*P*), the applied voltage (*V*), and resistance (*R*). As shown in Figure 7, the Joule heating properties of the textile heaters were examined. The temperature distribution of textile heater at different applied voltages was confirmed using an IR camera and FLIR software. Figure 7a shows the maximum temperature of the heater fabricated with of sample 1. The DC applied voltage was supplied to the textile heater through contact electrodes. The temperature is exhibited for an applied voltage of 1 to 3 V. The temperature increased with the increasing applied voltage and the temperature distribution was uniform. When the applied voltage was 3 V, sample 1 was operated with a maximum temperature of ~108 °C. The response time reaching 90% of maximum steady-state temperature of sample 1 was within 18 s at a bias of 3 V. Since the response time until the heaters reached a steady state temperature was an important parameter in evaluating the performance of the heater, the rapid thermal response was another favorable feature of the E-textiles in practical applications [37,38]. Figure 7d showed the time-dependent change in the temperature of sample 2 with a gradually increasing applied bias. Sample 2 exhibited impressive thermal properties with a maximum temperature to 138 °C. The response time also varied depending on the applied voltage, but it was found to be very fast within 20 s. As shown in Figure 7c,f, both IR images of the two-film heater present uniform temperature distribution.

**Figure 8:**
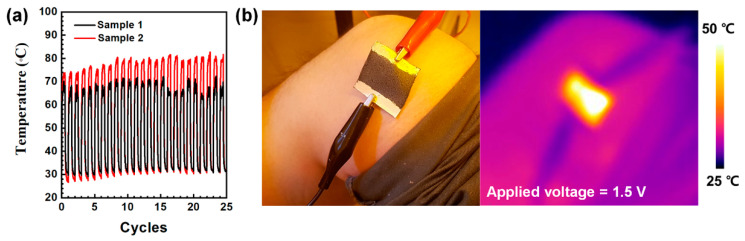
(**a**) Cyclic test of Joule heating properties of the E-textiles under a bias of 1.5 V and (**b**) the thermal patch application of sample 2 at the knee.

We finally investigated the long-term stability of Joule heating properties of the E-textiles. As shown in Figure 8a, the thermal properties of two textile heaters retained similar thermal evolution with low deviation during 25 cycles, which indicated a good operative stability of the textile heaters. The application of the E-textile in thermal therapy for uniform heat generation with low operating power can also be simply demonstrated, as shown in Figure 8b. We further investigated the long-term stability of the textile heaters by applying a bias of 2 V for 12 h in ambient condition. (Figure S5 in supplementary material) Sample 1 exhibited gradual decay of heat generation for initial 180 min and the thermal properties were completely lost after 480 min. In sample 2, more uniform heat generation of ~60 °C was retained for 120 min and thermal generation with a temperature >40 °C was retained for 720 min (Figure S6 in supplementary material). Although the thermal properties of the E-textiles fabricated in this work did not shown excellent stabilities, it would be possible to improve thermal/ambient stability for practical applications via further development of film properties and barrier films in the future.

## 4. Conclusions

In conclusion, we have demonstrated a straightforward way to prepare highly conductive E-textiles based on PEDOT:PSS via simple solvent treatment. We confirmed that the conductivity, morphology, and surface composition of PEDOT:PSS films is improved by immersion in methanol/DMSO solution, which resulted in significantly improved electrical conductivity of the PEDOT:PSS-coated E-textiles. The methanol/DMSO-treated E-textiles possessed not only superior electrical properties but also good operative stability in ambient condition. More importantly, it also endured washing and stretching tests without significant loss in conductivity, which could enable practical applications of the E-textiles such as wearable electronics. We also demonstrated the textile heaters with a low operative power and a short thermal response using the PEDOT:PSS-coated E-textiles. This work provides insights of highly conductive PEDOT:PSs-based E-textile by simple solvent treatment, which signifies the importance of design and processing of conductive polymers for realizing improved electrical performance and its E-textile applications.

## Data Availability

Not applicable.

## Supplementary Materials

The following are available online at https://www.mdpi.com/2073-4360/13/6/945/s1, Table S1: Elemental mapping analysis of E-textiles, Figure S1: Optical microscope images of E-textiles, Figure S2: Elemental mapping (EDS) of E-Textiles, Figure S3: SEM images of E-textiles (sample 1), Figure S4: SEM images of E-textiles (sample 2), Figure S5: Long-term assessment of textile heaters, Figure S6: IR images of textile heaters in long-term operation.
