# Supplementary material for: A Facile Solution Engineering of PEDOT:PSS-Coated Conductive Textiles for Wearable Heater Applications
Source: Polymers (Basel). 2021 Mar 19;13(6):945. doi: 10.3390/polym13060945 (PMC8003570; doi:10.3390/polym13060945)
Supplement: Supplementary file 1 [file polymers-13-00945-s001.pdf]

Supplementary data for

A Facile Solution Engineering of PEDOT:PSS-Coated Conductive Textiles for Wearable Heater Applications

In Su Jin [1], Jea Uk Lee [1,*] , and Jae Woong Jung [1,*]

[1] Integrated Education Institute for Frontier Science & Technology (BK21 Four), Department of Advanced Materials Engineering for Information and Electronics, Kyung Hee University, Yongin-si, Gyeonggi-do 446-701, Korea

*Correspondence: wodndwjd@khu.ac.kr (J. W. Jung); leeju@khu.ac.kr (J. U. Lee)

Table S1. Elemental mapping analysis of the E-textiles.

Sample 1			Sample 2		
Element	Weight (%)	Atomic (%)	Element	Weight (%)	Atomic (%)
C	57.16	69.65	C	61.04	72.48
O	26.99	24.69	O	26.18	23.34
S	11.71	5.35	S	8.73	3.88
Pt	4.14	0.31	Pt	4.05	0.30
Totals	100.00		Totals	100.00	

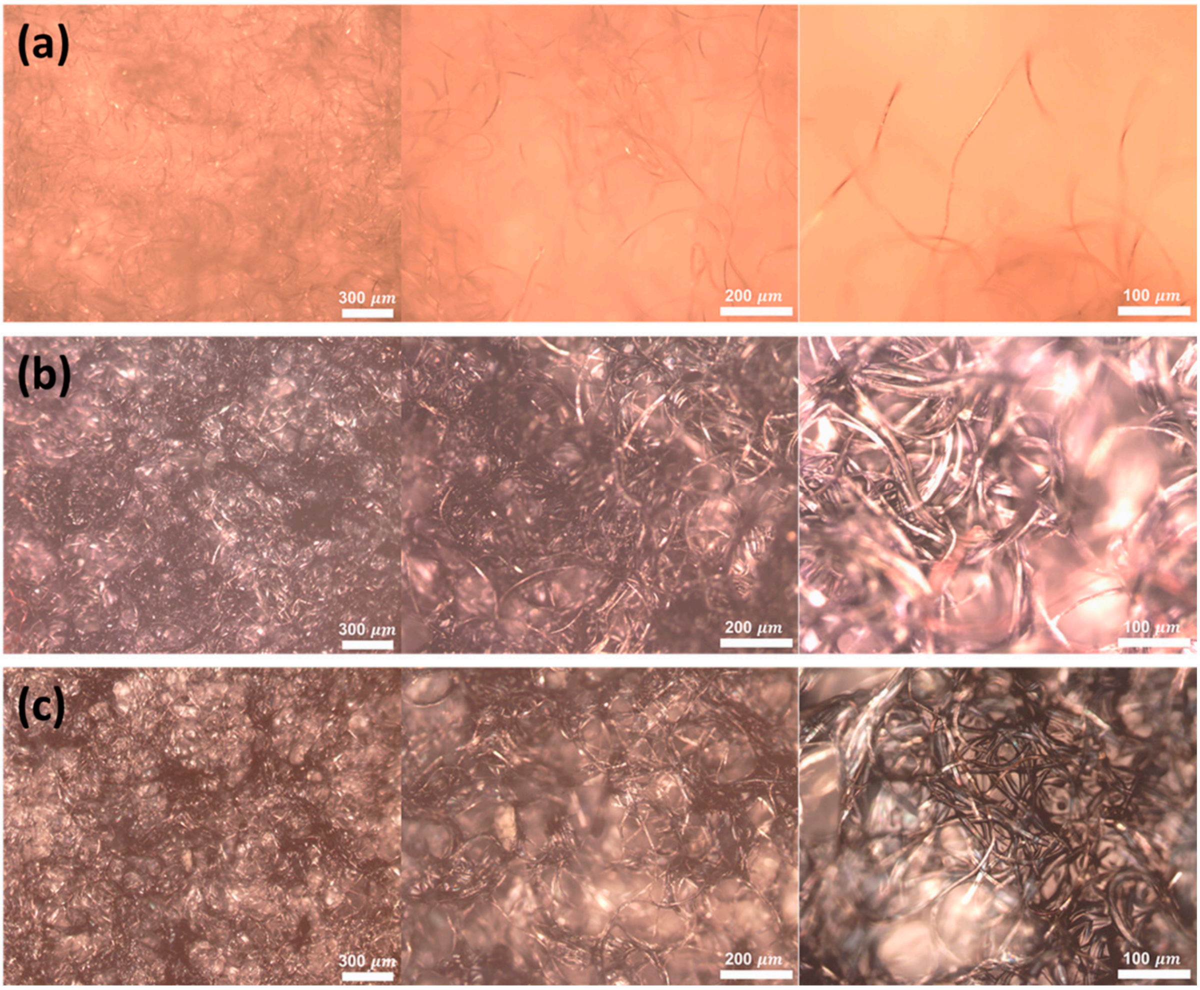

Figure S1. Optical microscope images of (a) pristine textile, (b) PEODT:PSS/DMSO-coated textile and (c) PEDOT:PSS-coated textile treated with methanol/DMSO solution.

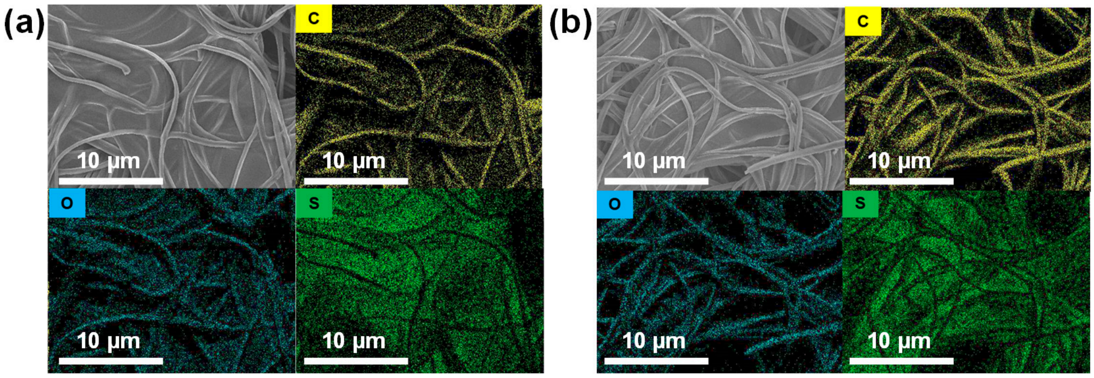

Figure S2. Elemental mapping images of carbon, oxygen, and sulfur on the surface of sample 1 (a) and sample 2 (b).

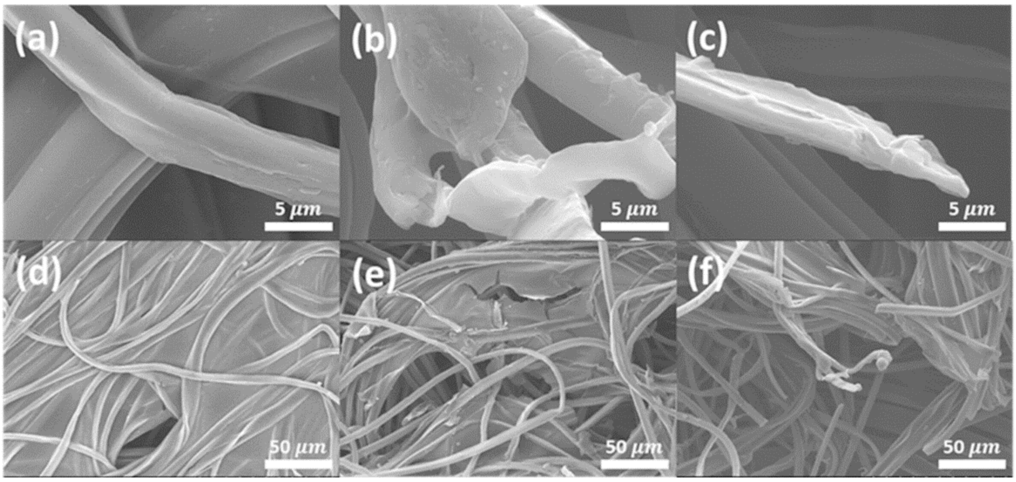

Figure S3. SEM images of sample 1 before stretching (a, d), during stretching (b, e), and after stretching (c, f).

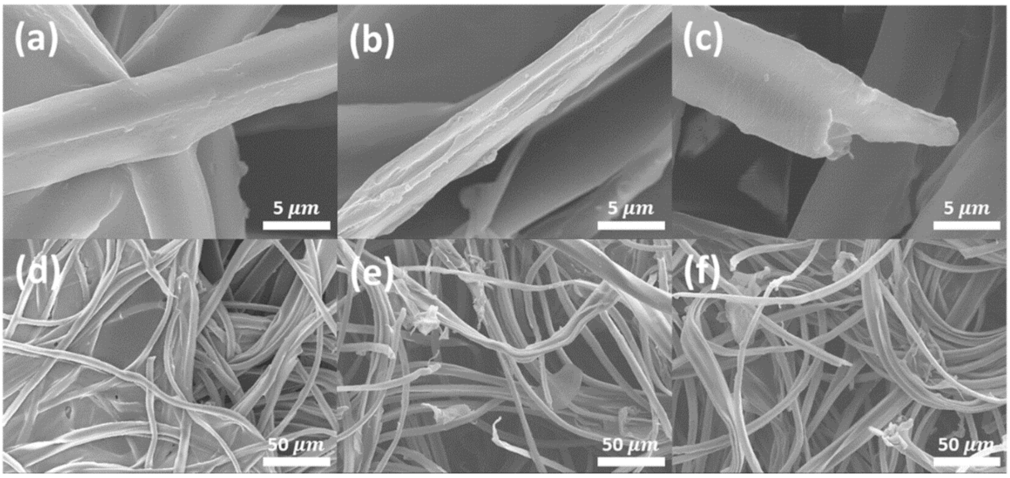

Figure S4. SEM images of sample 2 before stretching (a, d), during stretching (b, e), and after stretching (c, f).

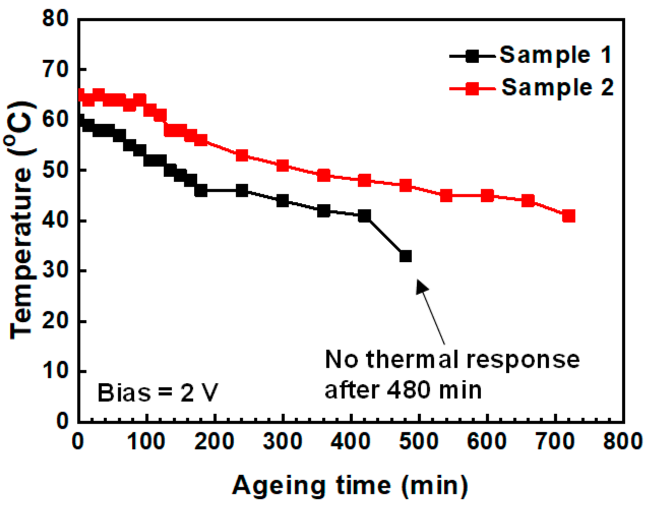

Figure S5. Long-term assessment of thermal property of E-textiles under 2 V bias.

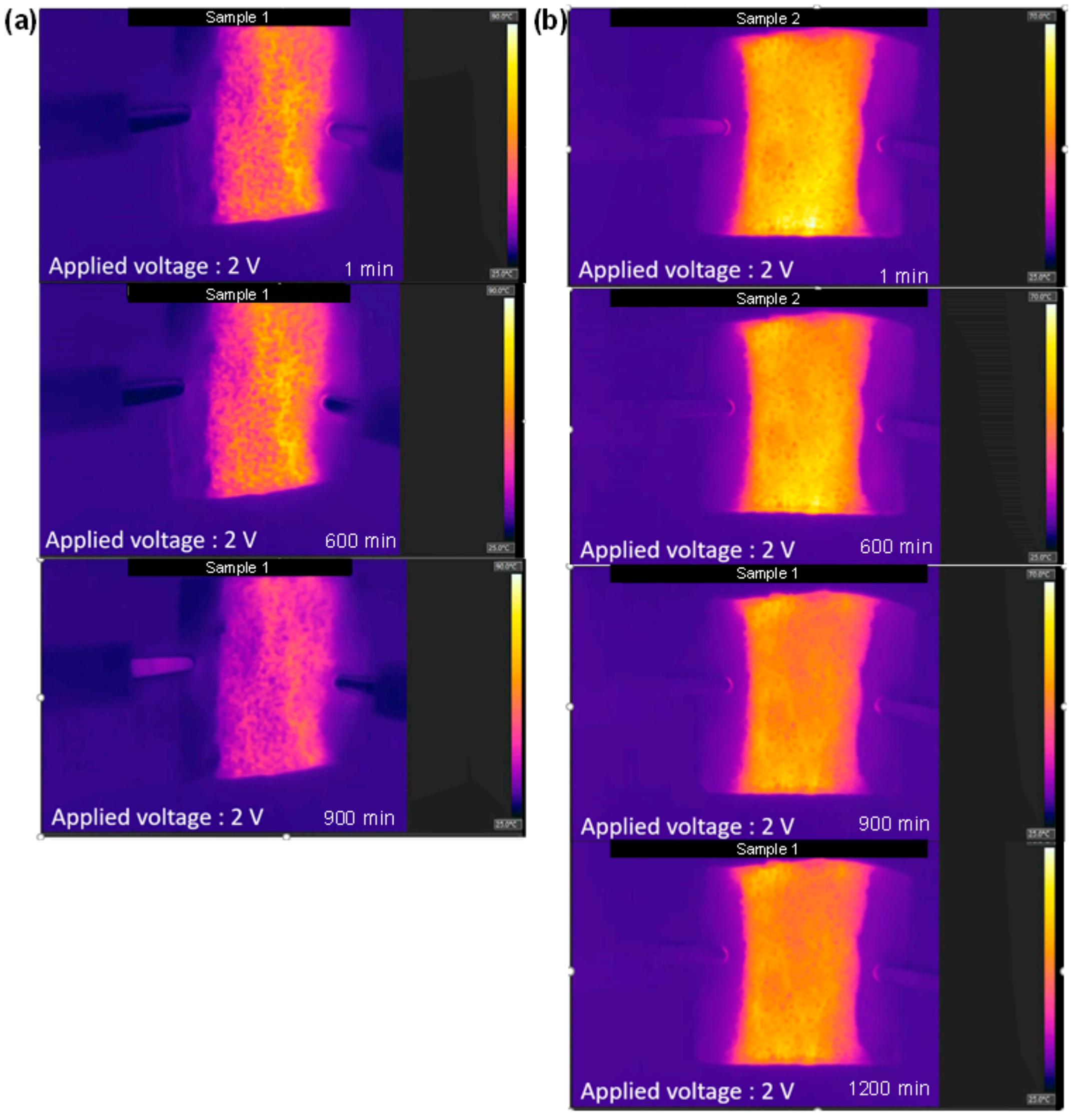

Figure S6. Infrared thermal images of E-textiles under long-term operation with 2 V bias.
